# An automated bacterial concentration and recovery system for pre-enrichment required in rapid *Escherichia coli* detection

**DOI:** 10.1038/s41598-018-35970-8

**Published:** 2018-12-13

**Authors:** Yushan Zhang, Chang-qing Xu, Tianyi Guo, Lingcheng Hong

**Affiliations:** 1 Faculty of Engineering, McMaster University, Hamilton, Canada; 2 Forsee Instruments Ltd., Hamilton, Canada; 3 Jiangsu Delin Environmental Protection Technology Co., Ltd., Jiangsu, China

## Abstract

One of the biggest challenges in rapid low concentration bacterial detection is the pre-concentration or pre-enrichment, which aims to increase bacteria concentration and reduce sample volume for easy bacterial detection. In practical bacterial detection, large-volume water samples with a pathogenic bacterial concentration of less than 1 CFU/mL have to be tested rapidly. The reported biosensors either have insufficient detection limit or have limited capability of handling a sufficiently large water sample. Therefore, a high-performance automated pre-enrichment process is strongly demanded in rapid practical bacterial detection. In this paper, a practical high performance automated bacterial concentration and recovery system (ABCRS) based on the combination of a ceramic membrane and tangential flow filtration technique was presented with short processing time (less than one hour), low pre-enrichment limit (≤0.005 CFU/mL), high concentration ratio (≥ 500), high recovery efficiency (~ 90%), and small final retentate volume (≤ 5 mL).

## Introduction

Rapid detection of pathogenic bacteria has attracted a lot of attention as it has enormous impact on public health. It is estimated that more than 2.2 million deaths per day are caused by the water contaminated with infectious diseases around the world^1^, and about 9.4 million illnesses and 1351 deaths every year in the United States are due to foodborne pathogen contaminated food^2^. In the drinking water and food industry, rapid bacterial detection methods that are capable of monitoring the pathogenic bacteria at low concentrations (e.g. <1 CFU/mL for *E*. *coli*) are vitally demanded to reduce risks to public health. Generally speaking, a rapid pathogenic bacteria detection system consists of five modules: pre-enrichment, capture, detection, signal transmission and data analysis^3^. The total processing time should include the length of time each necessary step takes, from the pre-enrichment step to the read-out of the results. For practical applications, the system must meet the following requirements: low detection limit (≤1 CFU/mL), fast processing (≤1 hour), and high specificity/portability/stability, as well as fully automatic^4–7^.

The current bacterial detection methods include cell culture, nucleic acid-based sensing, immunoassays and biosensors^8^. The standard microbiological culture method requires a few days to obtain the results, implying that this method is not appropriate for rapid bacteria detection. Furthermore, the total number of the cultured bacteria can be underestimated as some specific bacteria can enter a viable but non-culturable state in environment^9,10^. Polymerase chain reaction (PCR)-based assays are the most common nucleic acid-based techniques used in laboratory for rapid bacterial detection. Real time PCR-based assays have a detection limit of 10 to 100 CFU/mL for *E*. *coli* detection^11,12^. However, PCR usually needs at least 6 ~ 48 hours enrichment for low concentration bacterial detection^12–16^. In addition, expensive equipment and highly-trained personnel are required for the PCR-based assays. Rapid immunoassays, such as, enzyme-linked immunosorbent assays (ELISA) are also labor-intensive and the detection limit of ELISA is usually high (10^4^ ~ 10^6^ CFU/mL)^11^. This relatively high detection limit still needs to be lowered to monitor the pathogenic bacteria at low concentration in drinking water. Other than ELISA, lateral flow assay (LFAs) can be operated by non-specialized personnel^17,18^, and this method reduces the assay times from hours to minutes^19–21^. The detection limit of the LFAs is also limited by the bacterial concentration in initial water sample. Sample enrichment process is needed in many LFAs to improve the specificity and sensitivity^17,21^.

Recently, various detection techniques based on biosensors have been developed to reduce the assay time and to improve the sensitivity for rapid monitoring of the pathogenic bacteria. A label-free immune-based biosensor with a detection limit of 2 CFU/mL has been reported for detection of *E*. *coli*^22^. However, the flow rate used in the immunosensor detection was 56 µL/min. In other words, the sensors can only test a sample with an assay volume on nanoscale or microscale (i.e. µL ~ mL)^22^. Considering the fact that large volumes of initial water samples (100 mL ~ 1000 L) are required for the tests in food industries and environmental monitoring^23^, the total assay volume of these single-cell detection biosensors can handle is too small for practical rapid detection. Moreover, the bacterial indicators in recreational water should be less than 100 CFU/100 mL suggested by the United States Environmental Protection Agency (USEPA)^3^. Therefore, the assay volume in bacterial detection should be at least 1 mL so that at least 1 cell is included in the tested assay. As can be seen, a high-performance pre-treatment process is necessary to reduce the initial water sample from a relatively large volume to a small final assay volume (e.g. ~ mL) for single-cell detection biosensors in practical applications.

For the biosensors or lab-on-a-chip devices with a moderate detection limit (e.g. 10 ~ 10^4^ CFU/mL), the pre-enrichment process is also important since the target bacterial concentration in the assay needs to be increased^3,8,13,23–25^. As discussed below, in combination with a high-performance pre-enrichment process, biosensors with a moderate detection limit can be used to detect bacteria of low initial concentrations (e.g. ≤1 CFU/mL).

The reported pre-enrichment methods for rapid pathogen detection include microbial culture, centrifugation, immunomagnetic separation (IMS) and bacterial concentration. Bacterial concentration is the most appropriate method to concentrate the large amount of initial water sample to a few milliliters in a short time period. The use of hollow-fiber, NanoCeram filter, and porous anodic alumina membrane, as well as some other filters to recover multiple microorganisms from large amounts of water samples have been reported^26–31^. Hollow-fiber with tangential-flow ultrafiltration technique has been applied to concentrate and recover *E*. *coli* by Liu *et al*., resulting in a final volume of about 440 mL^32^. Usually, final volumes around 250 ~ 500 mL can be achieved by the hollow-fiber^32–41^. Thus, a secondary treatment as centrifugation has been reported to increase the concentration ratio and further reduce the final volume^38^. Furthermore, the reported recovery efficiency of pathogen is in a range of 42% ~ 96% depending on different filtration techniques^32–41^. Therefore, the reported methods still need to be improved to satisfy the requirements for rapid low concentration bacterial detection.

In this paper, an automated bacterial concentration and recovery system (ABCRS) as a high-performance pre-enrichment step is presented for rapid low concentration bacterial detection (≤1 CFU/mL). As the microbial indicator of water quality, *E*. *coli* was used to evaluate the performance of the ABCRS. The results show that the ABCRS can concentrate large amount of water sample (e.g. 1 ~ 2 liters) to a few milliliters (e.g. 4 ~ 5 mL) with an average recovery efficiency above 92% in less than one hour. The recovery efficiency of the ABCRS is studied in terms of the back-flushing technique, final retentate volume and volume concentration factor.

## Results and Discussions

### Effects of back-flushing on the ABCRS

**Figure 1:**
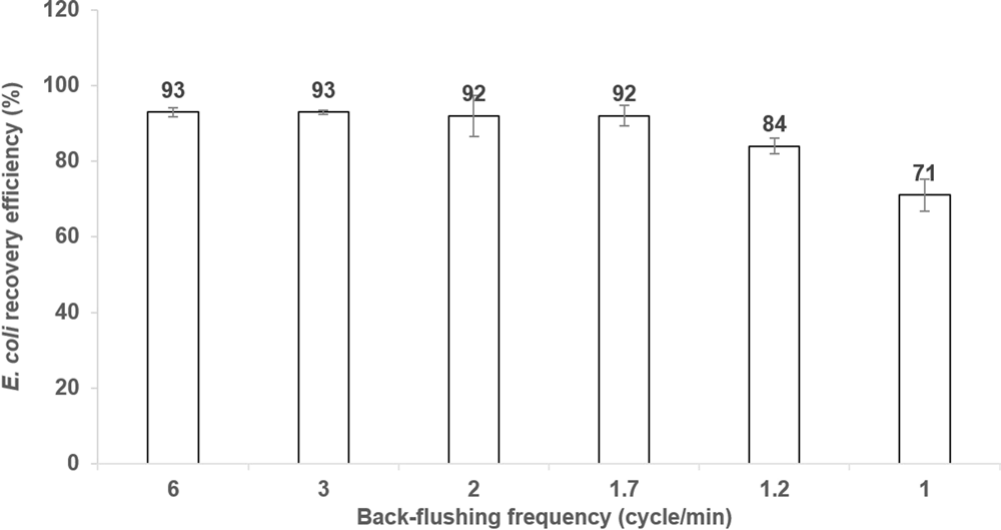
Measured *E*. *coli* recovery efficiencies of the ABCRS at various back-flushing frequencies.

As a key factor, recovery efficiency was used to evaluate the capability of the ABCRS to harvest the bacteria from an original large-volume water sample. Fig. 1 shows the measured effects of forward time and back-flushing frequency on the recovery efficiency. 100 mL solutions of known quantities of 10^5^ ~ 10^6^ *E*. *coli* cells were spiked to the sample reservoir and these samples were concentrated to about 5 mL by the ABCRS. The ABCRS with back-flushing frequency of 6 cycles/min recovered 93% of *E*. *coli* cells and the recovery efficiency remained stable as the back-flushing frequency dropped down to 3 cycles/min. A slight decrease in *E*. *coli* recovery efficiency was found when the back-flushing frequency decreased to 2 cycles/min. A significant decrease was noted at 1.2 cycles/min, resulting in an average *E*. *coli* recovery efficiency of 84% (as shown in Fig. 1). In contrast, a steep recovery efficiency decline was paralleled by a further drop in back-flushing frequency. The recovery efficiency was only 71% at a back-flushing frequency of 1 cycle/min. As a result, the back-flushing frequency was set at 2 cycles/min for subsequent experiments to achieve stable recovery efficiency and higher permeate flow rate. It is worth noting that the effect of back-flushing on increasing the recovery efficiency has also been reported^27,32,42^. In the previous studies, back-flushing was performed at the very end of the concentration process by adding extra solution (50 mL or more) to the retentate reservoir to wash the target micro-organisms off from the filters or membranes^33,43–45^. The total final volume was100 mL or more in these filtration techniques^37,43,44^, and this assay volume is much larger than that can be handled by most microfluidic based sensors. In this study, the back-flushing was used at the end of each forward flow in the filtration cycle, and this back-flushing method without elution-solution reduced the final volume collected at the retentate reservoir. The reduced final volume of milliliter-scale can be processed by microfluidic sensors^46^.

In the reduced final volume, the bacteria were blocked and captured by the ceramic membrane, and these bacteria cells needed to be transferred to the reservoir. The current study confirmed that back-flushing was effective for bacterial recovery and proved that a higher back-flushing frequency was more effective than a lower back-flushing frequency (Fig. 1). The reason for the increased recovery efficiency was that the back-flushing flow provided a reverse force from the bottom to the top of the ceramic membrane to wash the bacteria cells off from the membrane to the liquid flow. As a result, more bacteria cells were then transferred to the retentate reservoir. The optimized back-flushing methods in this study significantly reduced the final retentate volume to a milliliter scale and a stable recovery efficiency of higher than 92% was achieved simultaneously.

### Final retentate volume of the ABCRS

Subsequent experiments were carried out to determine if the final volume can be minimized to obtain a higher concentration factor while maintaining stable and high recovery efficiency. As described above, precise final volume can be obtained by the PLC and the liquid level sensor. At first, the final volume was set at 10 mL, and the concentration factor increased with the increase of initial sample volume. As shown in Table 1, the measured recovery efficiency of a 10 mL retentate was 95%, 87%, 91%, and 90%, corresponding to the concentration factor of 10, 25, 50 and 75, respectively. As the final volume reduced to 5 mL from 100, 500, and 1000 mL initial samples with 10^5^ ~ 10^6^ *E*. *coli* cells, the measured recovery efficiency was 94%, 94% and 90%, respectively. No significant difference of recovery efficiency was observed as the change of initial sample volume or final retentate volume. Obviously, the ABCRS can recover *E*. *coli* from different amounts of water samples to a small amount of water. In this case, the initial volume and final assay volume can be altered by the ABCRS to meet the volumetric requirements in practical bacterial detection for biosensors. In particular, the results also indicated that 1000 mL water sample with a concentration of 1 CFU/mL can be concentrated in less than one hour and then monitored by a biosensor with a detection limit of 200 CFU/mL. This pre-enrichment step provided by the ABCRS can largely extend the application of biosensors in drinking water quality monitoring.

**Table 1:** *E*. *coli* recovery efficiencies of the ABCRS at various final retention volumes.

Initial volume (mL)	Final volume (mL)	Recovery efficiency (%)	± SD (%)	± RSD (%)
100	10	95	5.6	5.9
250	10	87	6.0	6.9
500	10	91	3.9	4.3
750	10	90	11.5	12.7
100	5	94	4.1	4.4
500	5	94	1.6	1.7
1000	5	90	4.4	4.9

### Volume concentration factor of the ABCRS

In the pre-enrichment process, a higher volume concentration factor implies a lower detection limit for the rapid pathogen sensing system. Table 2 shows the dependence of the measured recovery efficiency of the ABCRS on the volume concentration factor. 2.1 × 10^7^ to 2.4 × 10^7^ *E*. *coli* cells were seeded to 200 mL, 400 mL, 1000 mL and 2000 mL of phosphate buffered saline (PBS), corresponding to the concentration factor of 50, 100, 250 and 500, respectively. An average recovery efficiency of 87% was calculated when concentrating initial sample of 200 mL to a final volume of 4 mL. The recovery efficiency was 107% as the concentration ratio was 100, and this percent greater than 100% was due to a relative standard deviation (RSD) of 9.3%. The recovery efficiency ranged from 87% to 107%, indicating that more than 87% cells in the sample reservoir were recovered and the performance of the ABCRS under high concentration ratio was verified.

**Table 2:** *E*. *coli* recovery efficiencies of the ABCRS at various volume concentration factors.

Concentration Ratio	Initial volume (mL)	Final volume (mL)	Recovery efficiency (%)	± SD (%)	± RSD (%)
50	200	4	87	0.5	0.6
100	400	4	107	9.9	9.3
250	1000	4	99	10.9	11
500	2000	4	86	8.3	9.7

As mentioned above, a large volume of water sample with extremely low bacterial concentration has to be analyzed rapidly in water monitoring. The result in Table 2 indicates that the ABCRS has the capability to concentrate relatively large volumes of bacterial samples (e.g. 2000 mL) to small assay volumes (e.g. 4 mL) with recovery efficiencies above 86%. These results further imply that an initial water sample of 1000 mL with a bacterial concentration of 1 CFU/mL can be detected within one hour based on the fact that it only takes a few minutes to test a 1 ~ 4 mL water sample for a biosensor with a moderate detection limit (e.g. 100 CFU/mL^47^). We therefore assume that a detectable initial bacterial concentration of 0.004 ~ 0.04 CFU/mL can be expected if the ABCRS is connected to a sensitive biosensor with a lower detection limit of 1 ~ 10 CFU/mL^22,48^.

### The ABCRS pre-enrichment of extremely low *E*. *coli* concentration

To test the performance limit of the ABCRS, pre-enrichment experiments were carried out for water samples of extremely low *E*. *coli* concentrations. 500 mL PBS containing 20 ~ 80 *E*. *coli* cells were concentrated to 10 mL, and the recovered cells were cultured and counted by the USEPA Method 1603 (Table 3). Even though the initial concentration levels were about 0.1 CFU/mL, an average recovery efficiency of 82% was achieved for these low-seed *E*. *coli* samples.

**Table 3:** Recovery efficiencies of extremely low *E*. *coli* concentration using the ABCRS (20 ~ 80 *E*. *coli* cells).

ABCRS (cells)	Method 1603 (avg. cells)	Initial concentration (CFU/mL)	Permeate (cells)	Recovery efficiency (%)
54	57	0.11	0	95
41	45	0.09	0	91
18	20	0.04	0	90
56	64	0.13	0	88
22	28	0.06	0	79
43	60	0.12	0	72
28	47	0.09	0	60
Average recovery efficiency (%)				82
±Standard deviation (%)				13
±Relative standard deviation (%)				15

Less than 20 *E*. *coli* cells were introduced to 500 mL PBS and then concentrated by the ABCRS. Due to the extremely-low concentration, the real numbers of *E*. *coli* cells were about 3 ~ 18 measured by the USEPA Method 1603 (Table 4). As shown in Table 4, the recovery efficiency varied between 50% and 100% with an average value of 73%. In the absence/presence pathogen detection, 2 out of 3 *E*. *coli* cells were successfully captured by the ABCRS with an initial bacterial concentration of 0.005 CFU/mL. Therefore, the minimum concentration limit of the ABCRS was 0.005 CFU/mL.

**Table 4:** Recovery efficiencies of extremely low *E*. *coli* concentrations using the ABCRS (<20 *E*. *coli* cells).

Method 1603 (avg. cells)	ABCRS (cells)	Permeate (cells)	Initial concentration (CFU/mL)	Recovery efficiency (%)
3	2	0	0.005	67
5	4	0	0.010	80
8	5	0	0.016	63
10	10	0	0.020	100
11	8	0	0.022	73
11	9	0	0.023	82
17	11	0	0.033	65
18	9	0	0.036	50
Average recovery efficiency (%)				73
±Standard deviation (%)				15
±Relative standard deviation (%)				20

As mentioned above, a large volume of water sample with *E*. *coli* O157:H7, as few as 10 cells may have to be handled and detected in practical water quality monitoring^49^. Biosensors that can detect 10 *E*. *coli* O157:H7 cells in 1000 mL water have not been reported. By using the pre-enrichment process enabled by the ABCRS, 5 bacteria cells in 1 liter can be captured and recovered in a few milliliters for further detection by a highly-sensitive biosensor.

## Conclusions

Substantial amounts of initial water samples with low bacterial concentration need to be monitored in practical bacterial detection. Conventional bacterial culture and other time-consuming pre-enrichment methods are still necessary to increase the bacterial concentration for detection and the overall assay time is strongly affected. As for sensitive single-cell detection biosensors, the assay volumes still need to be improved to handle substantial amounts of water samples^4,24,27,50^. With the aim of developing a fast and reliable pre-enrichment process for the existing biosensors and other devices to rapidly detect water samples of extremely low bacterial concentrations in practical situations, a high performance automated bacterial concentration and recovery system based on a ceramic membrane has been proposed.

The recovery efficiency of the ABCRS has been studied in terms of back flushing frequency, final retentate volume, volume concentration factor, and extremely low *E*. *coli* concentration Therefore, the ABCRS can satisfy all the requirements for practical rapid pathogen detection, i.e. fast (<1 h), small retentate final volume (<5 mL), high recovery efficiency (>92%), and large concentration factor (from >2000 mL to <5 mL). It has been found that the proposed ABCRS has capability to harvest 20 ~ 80 *E*. *coli* cells and less than 20 *E*. *coli* cells in a water sample volume of 500 mL with an average recovery efficiency 82%, and 73%, respectively. It is considered that, in combination with biosensors, the ABCRS will find profound applications in rapid pathogen detection for drinking water.

## Materials and Methods

### Sample preparation

#### Automated bacterial concentration and recovery system (ABCRS)

**Figure 2:**
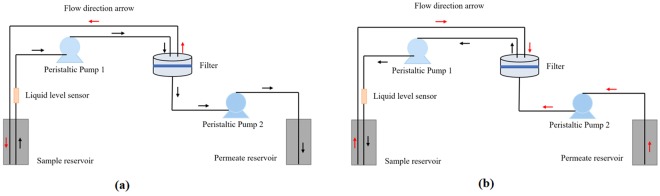
Schematic diagrams of an automated bacterial concentration and recovery system (ABCRS) with the tangential flow filtration technique used in the pre-enrichment process for (**a**) forward, and (**b**) backward flow procedure.

Figure 2 shows the schematic diagrams of the ABCRS with the tangential flow filtration technique used in the experiments for (a) forward, and (b) backward flow procedure. Fluid was pumped from a sample reservoir to a filter through a pump (peristaltic pump 1), running along the specially designed circular channels, and crossing a ceramic membrane surface inside the filter. The retentate flowed back to the sample reservoir and the permeate flow ran out to the permeate reservoir via another pump (peristaltic pump 2). The ABCRS was based on the tangential flow filtration technique which can efficiently recover the bacteria from water samples. The system consisted of two peristaltic pumps, a ceramic membrane holder, and two reservoirs for water sample and filtrate, as well as a liquid level sensor. PTFE (polytetrafluoroethylene) tubing with inner diameter of 1.6 mm and outer diameter of 3.2 mm was used to connect the ports in the flow system. Polyethylene bottles were used as the sample and retentate reservoirs. All these components were controlled by a programmable logic controller (PLC). The rotation direction and speed of the pumps, and the final volume can be controlled precisely by the PLC.

#### Concentration and recovery process

As shown in Fig. 2, the concentration process included two steps: the forward flow to filtrate the water sample (Fig. 2(a)), and the backward flow to reduce the bacterial accumulation on the ceramic membrane (Fig. 2(b)). A complete concentration and recovery cycle contained one forward flow process and one backward flow process. In the forward flow process, peristaltic pump 1 was mainly used to pump the water samples from the sample reservoir to the cylindrical filter holder, then peristaltic pump 2 guided the filtrate to the permeate reservoir. A recirculating flow ran back to the sample reservoir along the specially designed circular channels under a high pressure provided by the pumps. In the backward flow process, the reversal rotational direction of pump 2 provided a high-pressure permeate flow running back to wash the ceramic membrane. The concentration and recovery process can be stopped immediately when the designed final volume is achieved, and then the collected retentate can be transferred to the next step for detection.

#### Microorganism seeding

*E*. *coli* K-12 MG 1655 was cultured in tryptic soy broth at 37 °C for one night before the experiments. The cultured *E*. *coli* was centrifuged and re-suspended in PBS, then enumerated under microscope (Nikon Eclipse E200, Japan) before use. Re-suspended *E*. *coli* cells were diluted to different concentration levels for the following concentrating process.

#### Microfilter set-up

A ceramic membrane (Sterlitech Corp., USA) was used in all experiments. Membrane diameter was 47 mm and the pore size was 0.14 µm. The ceramic membrane was kept in a stainless-steel cylindrical holder with a specially designed circulating channel (Fig. 2). 70% ethanol was injected to the flow system for sterilization after each run, then the system was rinsed with DI water serval times before next use. The membrane can be reused after cleaning with alkaline solution (sodium hydroxide) and acid solution (nitric acid) respectively.

#### Manual E. coli cells determination

Microscope counting with a hemocytometer (Bright-Line Hemacytometer, Hausser Scientific, USA) was used to determine the *E*. *coli* cells in the final volume obtained after concentration process for the concentration ranging between 10^5^ and 10^6^ CFU/mL. For low-seed *E*. *coli* samples, contrast groups containing the same amounts of *E*. *coli* were monitored by the USEPA Method 1603 directly. The experimental groups containing the same numbers of *E*. *coli* were first spiked to either 500 mL or 1000 mL PBS to run through the ABCRS, and then the *E*. *coli* cells recovered in the final retentate were measured by the USEPA Method 1603.

### The pre-enrichment of the ABCRS

The above described ABCRS was studied and optimized based on four important parameters: back-flushing condition, volume concentration factor, final volume and recovery efficiency.

#### Back-flushing

A backwashing procedure (the alternative working of the forward and backward flows) was set at the end of each forward flow process, instead of at the very end of the filtration process. The duration of the back-flow process was fixed at 1 second (s), while the duration of forward flow process was set at different values in subsequent experiments. By alternating the rotation direction of the peristaltic pumps, the forward flow procedure and backward flow procedure worked alternatively at a specific frequency controlled by the PLC. The timer of backward flow procedure was set at 1 s and the timer of forward flow procedure was set at 10 s, 20 s, 30 s, 40 s, 50 s and 60 s in the experiments, which corresponded to a backwashing frequency of 6, 3, 2, 1.7, 1.2 and 1 cycle(s)/min, respectively. *E*. *coli* samples of 100 mL with known concentration levels ranging from 2 × 10^4^ ~ 2 × 10^5^ CFU/mL were concentrated to about 5 mL at various back-flushing frequencies to study the effect of the backwashing on *E*. *coli* recovery efficiency.

#### Final volume

A liquid level sensor (Fig. 2) located on a tube between the sample reservoir and peristaltic pump 1 was used to control the final retentate volume. During the concentration process, the tube was filled with water and the sensor started to monitor the liquid level. Once air bubbles were observed, the liquid level sensor sent a signal to the PLC to stop the process. By adjusting the position of sensor and the diameter of the tube, a specified retentate volume (final volume) was achieved. In this research, final volumes were set at 10 mL, 5 mL and 4 mL, respectively, to satisfy the typical assay volume requirement for the subsequent process of bacterial detection.

#### Volume concentration factor

The volume concentration factor (C) was defined as the ratio of the initial volume of the water sample to the final retentate volume. In the experiments, *E*. *coli* samples with an initial volume of 500 mL, 1000 mL and 2000 mL were concentrated to a final volume of 10 mL and 5 mL to test the sensitivity and reliability of the ABCRS.

#### *E. coli* absence/presence detection

To evaluate the performance of the ABCRS for *E*. *coli* absence/presence detection, original *E*. *coli* samples were diluted to 5 ~ 150 CFU/mL according to the standard dilution process suggested in the USEPA Method 1603. In absence/presence experiments, 5 ~ 150 *E*. *coli* cells were seeded in 500 mL PBS to run through the ABCRS and then the recovered *E*. *coli* cells in the retentate were measured by the USEPA Method 1603. In the contrast groups, the 5 ~ 150 *E*. *coli* cells were counted by the USEPA Method 1603 directly.

### Calculation and data analysis

The recovery efficiency of each test was calculated as the ratio of the total number of *E*. *coli* in the final retentate to the total number of *E*. *coli* in the initial water sample. The volumetric concentration ratio (C) of individual test was calculated by the ratio of initial sample volume to the final retentate volume. All the experiments of back-flushing, final volume, and volume concentration factor sections were repeated for more than five times. The average recovery efficiency and standard deviation of these repeated tests were used to evaluate the performance of the ABCRS. For *E*. *coli* absence/presence detection, the range of the total *E*. *coli* cells were controlled to 20 ~ 80 cells and less than 20 cells in 500 mL PBS. Each test was done once due to the extreme-low number of the bacteria cells.
